# Evaluation of Holstein cows with different tongue-rolling frequencies: stress immunity, rumen environment and general behavioural activity

**DOI:** 10.1186/s40104-023-00906-4

**Published:** 2023-08-11

**Authors:** Fuyu Sun, Xiaoyang Chen, Yongfeng Li, Guangyong Zhao, Xianhong Gu

**Affiliations:** 1 State Key Laboratory of Animal Nutrition, Institute of Animal Science, Chinese Academy of Agricultural Sciences, Haidian District, No.2 Yuanmingyuan West Road, Beijing, China; 2 College of Animal Science and Technology, China Agricultural University, Haidian District, No.2 Yuanmingyuan West Road, Beijing, China; 3 Agricultural Information Institute, Chinese Academy of Agriculture Sciences, Haidian District, No.12 Zhongguancun South Street, Beijing, China

**Keywords:** General activity, Holstein cow, Rumen fermentation, Stress immunity, Tongue rolling

## Abstract

**Background:**

The tongue-rolling behaviour of cows is regarded as an outward sign of stressed animals in a low welfare status. The primary aim of this observational study was to evaluate the association between the frequency of tongue-rolling behaviour and its physiological function. The secondary aim was to explore the relationship between general activities and the frequency of tongue-rolling behaviour of cows. A total of 126 scan sampling behavioural observations were collected over 7 d on 348 Holstein cows with the same lactation stage in the same barn. The tongue-rolling frequency was defined as the number of tongue-rolling observations as a percentage to the total observations per individual cow. According to their tongue-rolling frequency, the cows were grouped into the CON (no tongue-rolling), LT (frequency 1%), MT (frequency 5%), and HT (frequency 10%) groups. Six cows from each group were randomly selected for sampling. Serum samples, rumen fluid, milk yield, and background information were collected. The general behaviour data during 72 continuous hours of dairy cows, including eating time, rumination time, food time (eating time + rumination time), and lying time, were recorded by the collar sensor.

**Results:**

Cortisol (*P* = 0.012), γ-hydroxybutyric acid (*P* = 0.008), epinephrine (*P* = 0.030), and dopamine (*P* = 0.047) levels were significantly higher in tongue-rolling groups than in the CON group. Cortisol levels and tongue-rolling frequency had a moderate positive correlation (linearly *r* = 0.363). With the increase in tongue-rolling frequency, the rumen pH decreased first and then increased (*P* = 0.013), comparing to the CON group. HT cows had significantly less food time than CON cows (*P* = 0.035). The frequency of tongue-rolling had a moderate negative relationship with rumination time (*r* = −0.384) and food time (*r* = −0.492).

**Conclusions:**

The tongue-rolling behaviour is considered as a passive coping mechanism, as the stress response in cows with high tongue-rolling frequency increased. Food intake and rumination activities were all closely related to the occurrence of tongue-rolling behaviour.

## Introduction

**Fig. 1:**
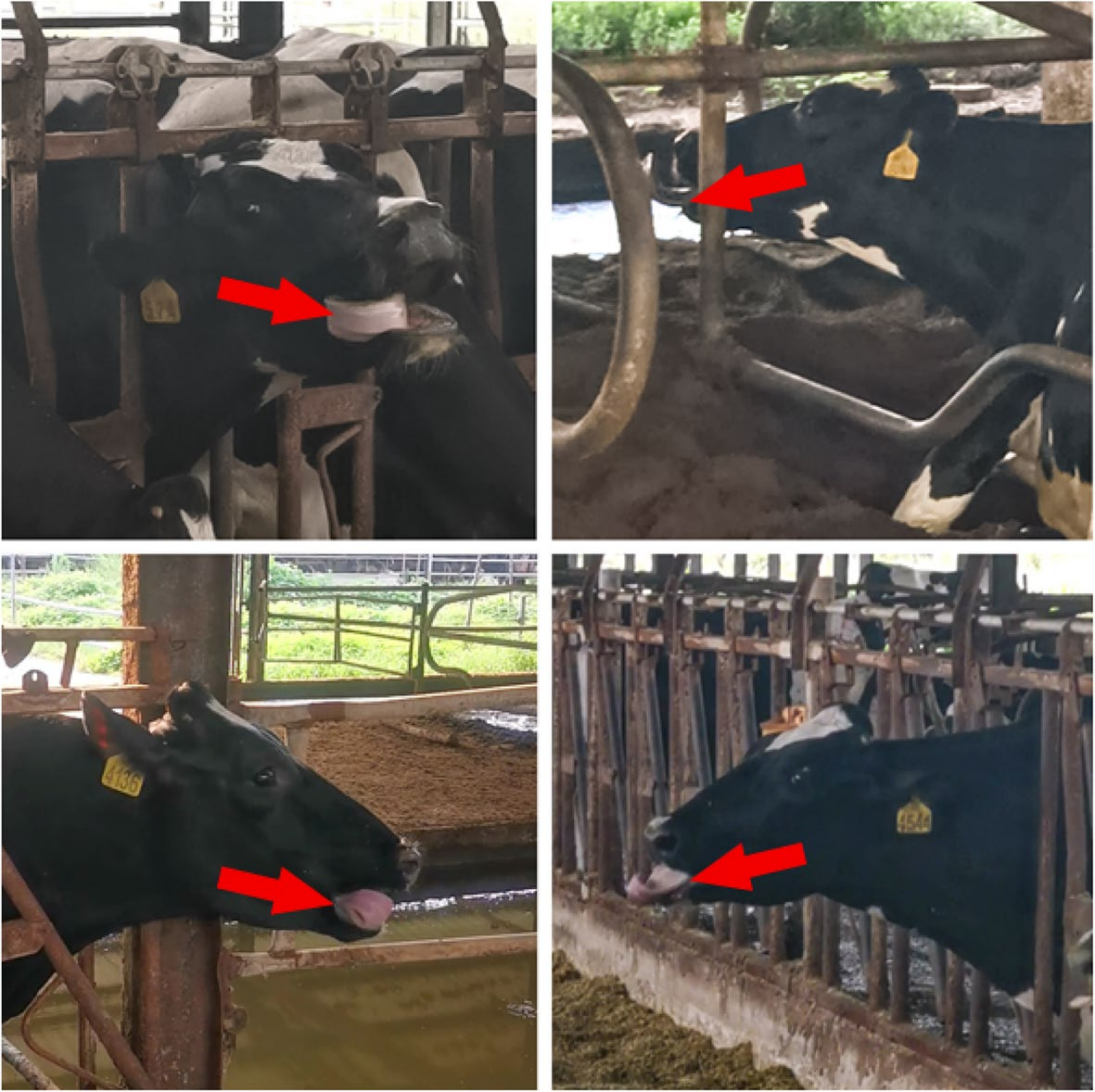
Tongue rolling behaviour of Holstein cows (photos taken in the experimental study)

Stereotypical behaviour was observed to occur in domesticated species and other animals kept in captivity [1]. Stereotypic behaviour, especially non-nutritive oral behaviour, was observed in indoor-farmed dairy cows at all growth stages, regardless of the local climates [2, 3]. Among them, tongue rolling was a typical stereotypical behaviour, manifested as non-functional and repetitive. The clinical symptoms was: the tongue remains in a fully or partially rounded state, quickly moving from side to side [4]. The behaviour might occur within the boundaries of the inner lips or stretch to the outer boundaries of the lips (Fig. 1). In a previous study, we found that tongue-rolling behaviour was a common stereotype that 18% of the domestic cows showed this behaviour [5]. The researchers hypothesized that this behaviour might be related to feeding patterns. In the grazing environment, cows yank off the naturally growing forage with their tongue, which is the so-called tearing behaviour. While the feeding mode was changed in the captive environment, which hindered the cows from tearing behaviour [6, 7]. The low roughage ratio and quality TMR feed leads to decreased rumination time and poor rumen environment with subsequent changes in rumen microbes [7]. Increasing roughage level in TMR feed reduces the non-nutritive oral stereotypes, and vice versa [8]. And the tongue-rolling behaviour would not occur at the same time as other tongue behaviours, such as eating, ruminating, or licking non-nutritive substances [9]. Downey et al. [9] found that feeding hay to calves reduced their abnormal oral behaviours including tongue rolling and foodless regurgitation. Therefore, tongue rolling behaviour might be an alternative or functional oral behaviour to compensate for the lack of tongue activity or to relieve rumen discomfort [10].

The management of farms, production, genetic breeding, and animal welfare could be improved by focusing on cow behaviour. When animals were restricted or in low-welfare settings, some would have a negative mental state to cope with external environmental stress [11]. In the depression state, some of them exhibit stereotyped responses, which might be similar to those seen in low-stress situations, but with strong cortisol and catecholamine reactions when detecting their hypothalamic–pituitary–adrenal (HPA) axis and locus coeruleus-norepinephrine (LC-NE) system [12, 13]. The frequency of individual stereotyped behaviour was associated with plasma cortisol and adrenocorticotropic hormone levels in calves and young cattle [14]. The intensity of stereotyped behaviour (including the frequency and duration) might be correlated with the stress level. Cows that express oral stereotyped behaviour at high frequencies exhibit high levels of stress response and metabolism, suggesting that oral stereotyped behaviour might be an adaptive behaviour that relieves stress [14]. Our previous study found that cows with high tongue-rolling behaviour frequency had lower ruminal pH and more lying and drinking time, which are important general activity indicators, as well as higher stress levels than normal cows [5]. Although these behaviours were frequently stereotyped in form, it was hypothesized that they serve an adaptive purpose [15]. According to Freymond's findings [16], some stereotyped behaviour might be a coping mechanism that enables stereotypical individuals to lower cortisol levels [17]. The stereotyped behaviours enable animals in the barn to stabilize the stress response and to reduce or eliminate their discomfort, which was to some extent an adaption to the low welfare environment. But this conclusion has not been verified in the tongue-rolling behaviour of lactating cows.

Thorough monitoring was crucial for the early detection, investigation, and management of stress [18]. The duration and intensity of unitary behaviours and movements of cattle also help predict their physical states, such as calving, estrus, lameness, and disease [19]. Visual locomotion scores for different parts were the simplest form of on-farm sickness, lameness, and disease detection [20]. Van Nuffel et al. [21] pointed out that although some scoring methods are relatively easy to use, it is time-consuming to score the entire herd. Besides, the inappropriate attitude or behaviour of farmers might frighten the herd and reduce productivity [22]. Sensor-based technology is gradually replacing the labour- and time-intensive traditional visual inspection method [23]. Wearable sensors were among the most commonly designed and well-accepted due to their real-time monitoring of cattle behaviours [24]. Accelerometers or inertial measurement units (IMU) sensors can quantify changes in force during the postural shift and local movement to identify unitary behaviours [25, 26]. The majority of studies used time changes in cows' general behaviour (single or multiple general actions) to determine their physiological and welfare status. It has been reported that 3-axis accelerometers or IMU with a gyroscope attached to a cow's neck or ear could identify its unitary activities, including feeding, ruminating, standing, lying, and walking [27, 28]. However, stereotyped behaviours may disrupt their normal general activities. Therefore, the correlation between the general activities of dairy cows and the occurrence of stereotyped behaviours needs to be studied.

Previous studies compared the indicators of cows with and without high-frequency togue-rolling behaviour [5], while the function and trend of different frequency tongue-rolling behaviours were not yet clear. Moreover, information on the association between the frequency of tongue rolling, stress, physiological indicators, and general activity behaviour in cows was not sufficient. Therefore, our objective was to assess the differences in stress indicators and changes in rumen fermentation function in cows with different tongue rolling frequencies. The second objective was to investigate the effect of different tongue rolling frequencies on the duration of general behaviour in cows.

## Materials and method

### Animals and management

The experiment was performed at the cow farm of the Shandong Yinxiang Weiye Group Company (Cao County, Shandong Province, China, 34°82′N, 115°54′E) from May 2021 to June 2021. A ventilated free-stall barn held 348 cows. The barn includes 2 pens and 400 stalls (about 174 cows per pen). The cows have free access to the outside, which was limited during the hot summer months, but they can still see it. With 1.15 stalls per cow, the indoor stocking density was 15.40 m^2^ per cow. Fans and sprinklers were installed in the cowshed for cooling. Cows were fed a total mixed rations (TMR) at 8:00, 15:30, and 20:00 ad libitum after being milked 3 times a day. Each round of milking took 20 min. The TMR was formulated according to the minimum nutritional requirements for high-producing dairy cows (NRC, 2001) [29]. Table 1 lists the dietary components and their nutritional value. An automatic manure scraper system was installed in the cow barn and the bedding for dairy cows was made from recycled manure solids. The bedding was changed and the cow barn was properly cleaned once a week to maintain hygienic cleanliness. The health state of the cows was evaluated by veterinarians every week.

**Table 1:** Ingredients and nutrient composition of experimental diets (%, DM basis)

Item	Content, %
Ingredients	
Alfalfa	10.39
Oat hay	2.42
Dandelion	0.48
Whole corn silage	48.33
Cottonseed	2.90
Beet pulp	2.42
Ground corn	7.49
Pressed corn	9.42
Soybean meal	8.70
Rapeseed meal	1.69
DDGS	0.72
Extruded soybean	1.33
Mineral and vitamin mix^1^	3.70
Nutrient composition	
DM, % of wet TMR	62.40
CP	17.06
EE	3.32
NDF	35.75
ADF	18.20
NE_L_, MJ/kg	6.11

*DM* Dry matter, *CP* Crude protein, *EE* Ether extract, *NDF* Neutral detergent fiber, *ADF* Acid detergent fiber, *TMR* Total Mixed Ration, *NE*_*L*_ Net energy for lactation, *DDGS* Distillers dried grains with solubles

^1^Mix contained the following per kg of diets: VA 170,000 IU, VD 8,000 IU, VE 19,000 IU, Ca 160 g, P 50 g, Fe 800 mg, Cu 680 mg, Mn 3,500 mg, Zn 7,500 mg, Se 80 mg, I 400 mg, Co 38 mg

### Experimental design and treatments

This study sampled cows with tongue-rolling behaviour and cows without abnormal behaviour and scanned 369 cows for observation (recording cow activity at pre-selected time intervals) [5]. Two well-trained observers recorded the behaviour of Holstein cows with the second lactation in the same barn (days of milk production = 153 ± 21, mean ± standard deviation). The behaviour observation period lasted 7 d. The observers first observed and evaluated how the same herd of cows behaved on the farm for 3 d (during the daytime). By calculating prevalence-adjusted, bias-adjusted kappa (PABAK) [30], the interobserver reliability highlights an almost perfect agreement of the abnormal behaviour assessment (PABAK > 0.8). The 6 h (8:00–11:00 and 14:00–17:00) with the highest prevalence of tongue-rolling behaviour in lactating cows were chosen based on the findings of the 3-day preliminary observation. We used the scanning sampling method for behaviour observation, recording the cow’s activities at a preselected time interval. The dairy cows were observed 126 times during the 7-d observation period, 6 h per day, 3 times per hour and 10 min per observation. Two observers walked slowly through the barn (200 m long) with a walking pace of 0.33 m/s to monitor the cows during each round. The observers were in the feeding channel and kept at least 2 m away from the observed herd to minimise interference with the cattle during the observation. Tongue rolling and other aberrant behaviours were noted by the cows' ear tag numbers. In 126 observations, the number of cows who rolled their tongues was counted and assessed. According to the observation logs for 7 d, there were a total of 99 cows with tongue-rolling behaviour, and the frequency of tongue-rolling behaviour observed was between 1%–10%. Six cows were randomly chosen from the low (1%), medium (5%), and high (10%) tongue-rolling frequency groups (LT group, MT group, HT group, more than 20 tongue-rolling cows per group), respectively. Six of the normal cows without togue-rolling behaviour were chosen as the control group. The selected cows had no other abnormal behaviour or disease symptoms. The selected cows were kept in their original pen and group-marked by veterinarian crayons of distinct colours.

**Fig. 2:**
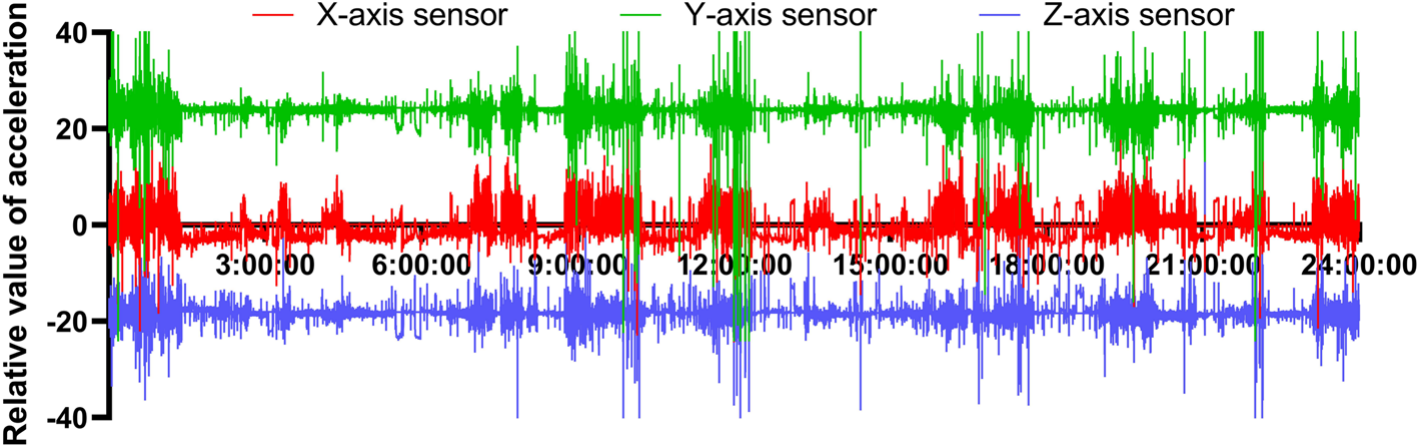
Example of *xyz* triaxial accelerator signal visualization results

In this study, to monitor daily eating, ruminating, and lying time, electronic collars (CowManager, Agis Automatisering BV; validated by Pereira et al. [31] in cattle farm) were attached to the cows' necks while milking. The neck-mounted IMU sensors (a 3-axis accelerometer and a 3-axis gyroscope) were used to collect motion data from cows (HOBO Pendant G Acceleration Data Logger, Onset Computer Corp.; validated in dairy cattle) [32, 33]. Accelerometers were set to record the g-forces of the *x*-, *y*- and *z*-axes at 40 s intervals. Data were automatically downloaded to a server from the devices by the installed cloud readers. The visualization results of the 3-axis (*x*, *y*, and *z*) accelerator signal are shown in Fig. 2. The four unitary activities including feeding, lying, ruminating, and standing were identified with synchronized recordings. Behaviour features were retrieved over four-time frames with 50% overlap, and 3 machine-learning methods were created to characterize unitary behaviours. The construction and behaviour recognition algorithm of the collar sensor were described by Li et al. [34]. During the experiment, the collar data were collected for 3 consecutive days. Devices were removed from the cows after the experiment. In addition, seven infrared surveillance cameras (Hikvision Digital Technology Co., Ltd., Hangzhou, China) were used to capture the action of cows for successive days (as a check and backup of collar data).

### Sampling and analysis

Tail vein blood was collected into 5-mL vacutainers (BD vacutainers, Fisher Scientific, Waltham, MA, USA) at 06:00 on a penultimate day after the end of the experiment and centrifuged at 3,000 × *g* for 15 min at 4 °C to separate serum. The serum samples were stored at −80 °C until assayed. The AU480 auto-analyzer (Olympus Co., Shinjuku, Toyko, Japan) was used to measure the serum concentration of lactate dehydrogenase (LDH), creatine kinase (CK), alanine aminotransferase (ALT), aspartate aminotransferase (AST), total protein (TP), and albumin (ALB). To measure cortisol (COR) in serum samples, a BFM-96 multi-tube radioimmunoassay counter (Hefei, Anhui, China) was utilized. Dopamine (DA), 5-hydroxytryptamine (5-HT), epinephrine (E), norepinephrine (NE), γ-hydroxybutyric acid (GABA), heat shock protein 70 (HSP-70), interleukin 6 (IL-6), interleukin 10 (IL-10), immunoglobulin A (IgA), immunoglobulin G (IgG), and immunoglobulin M (IgM) levels were measured by ELISA assays according to the manufacturer's instructions and data were read by Thermo Multiskan Ascent (Waltham, MA, USA).

On the final sampling day, 2 h after the morning feeding, ruminal fluid samples were collected using an oral stomach tube sampler. The first 100 mL liquids were discarded to avoid saliva interference. The following 50 mL of ruminal fluid were filtered through four layers of gauze with a 250-mesh size. The ruminal fluid pH was immediately detected using a portable pH meter (PB-10, Sartorius, Gottingen, Germany). To measure rumen volatile fatty acids (VFA) and ammonia-N (NH_3_-N), 20 mL of the ruminal fluid were mixed with 0.4 mL of 50% sulfuric acid and stored at −80 °C. Individual VFA and total VFAs were isolated and quantified using gas chromatograph (GC-2010, Shimadzu, Kyoto, Japan). The concentration of NH_3_-N was determined with a microplate assay. The rest of the rumen fluid samples were stored in liquid nitrogen.

### 16S rRNA gene sequencing in ruminal fluid

The microbial DNA was extracted from ruminal fluid samples [5], and 16S rRNA sequencing was conducted using the Illumina HiSeq 4000 platform (Illumina Inc., San Diego, CA, USA).

The microbiome data were analysed using QIIME2 (https://docs.qiime2.org/2019.7/tutorials/overview/). The operational taxonomic units (OTUs, 97% similarity cutoff) were grouped using UPARSE. Annotated taxonomy data from the ribosomal database project (RDP) classification system serves as the foundation for the Greengenes database (http://greengenes.lbl.gov). The RDP classifier used a 97% confidence criterion to link taxonomy with the SILVA (SSU115) 16S rRNA database (http://www.arb-silva.de).

### Statistical analysis

The information of body condition score, parities, and milk yield before the first day of the sample period was included as a covariate in the statistical analysis of each serum variable. Statistical data were analysed by 4-way ANOVA test for HT, MT, LT, and CON using the SPSS 21.0 software (SPSS, Chicago, IL, USA). To accomplish post-hoc pairwise comparison, multivariate Duncan tests were used. The results were presented as the mean values and SEM.

Spearman’s rank correlation analysis was applied to determine the relationships between tongue rolling frequency characteristics with serum stress indicators, rumen environment, and behavioural activity. *P*-values < 0.05 were considered statistically significant, and 0.05 < *P* < 0.10 were considered tendencies in all analyses.

Alpha diversity indices of the bacterial communities were assessed to obtain an overview of their richness and diversity through Chao1, Shannon, Simpson, ACE, and Good’s coverage indices. Beta diversity analysis was used to assess variations in sample phylum complexity. To compare the bacterial profiles between the groups, PCoA based on unweighted UniFrac distance metrics was carried out.

## Results

### Serum physiological, biochemical and immune indexes

Results of the serum stress parameters were reported in Table 2. The levels of GABA in the LT, MT, and HT groups were significantly higher than in the CON group (*P* = 0.008), but not significant between the 3 groups. COR concentrations in the HT and LT groups were significantly higher than in the CON group (*P* = 0.012). E concentrations in the MT and LT groups were significantly higher than those in the CON group (*P* = 0.012), and DA concentrations in MT groups showed a significant trend higher than that in CON group (*P* = 0.067).

**Table 2:** Differences on serum stress parameters among four group cows

Items	Experimental treatments^1^				SEM^2^	*P*-value
	HT	MT	LT	CON		
Cortisol, ng/mL	16.116^a^	13.988^ab^	15.509^a^	11.095^b^	2.074	0.012
HSP 70, pg/mL	42.213	46.124	45.025	46.204	1.634	0.706
γ-hydroxybutyric acid, μmol/L	1.586^a^	1.679^a^	1.672^a^	1.420^b^	0.052	0.008
Epinephrine, ng/mL	3.704^ab^	3.919^a^	3.894^a^	3.518^b^	0.094	0.030
Norepinephrine, ng/mL	17.860	18.863	18.637	16.659	0.650	0.502
5-hydroxytryptamine, pg/mL	510.303	522.812	525.122	484.348	11.749	0.538
Dopamine, nmol/L	24.429^ab^	25.990^a^	25.206^ab^	23.178^b^	0.686	0.067

^1^*HT* High frequency tongue rolling cows, *MT* Medium frequency tongue rolling cows, *LT* Low frequency tongue rolling cows, *CON* Control cows, no abnormal oral behaviour

^2^*SEM* Standard error of the mean

^a^^–^^c^Different superscript within a row means significant different (*P* < 0.05)

The results of serum immunity and inflammatory indicators were reported in Table 3. The IgA levels in each group increased first and then decreased compared with the CON group (*P* = 0.008). The levels of IgA in the MT group were significantly higher than in the other groups. The IgG levels in the 3 tongue-rolling groups were significantly lower than those in the CON group (*P* = 0.013). The levels of ALB in the LT and HT groups were significantly lower than those in the CON group (*P* = 0.049). There were no significant differences in other serum indicators.

**Table 3:** Differences in serum immunity and inflammatory indicators among four group cows

Items	Experimental treatments^1^				SEM^2^	*P*-value
	HT	MT	LT	CON		
IgA, μg/mL	621.296^b^	676.315^a^	631.422^b^	625.498^b^	10.609	0.008
IgG, mg/mL	19.951^b^	21.161^b^	20.552^b^	23.062^a^	0.483	0.013
IgM, mg/mL	5.382	5.603	5.422	5.676	0.105	0.278
Total protein, g/L	78.513	75.118	76.263	71.046	2.409	0.202
Albumin, g/L	28.272^b^	30.323^ab^	29.780^b^	33.814^a^	1.354	0.049
IL–6, ng/L	434.009	461.247	444.857	423.928	14.562	0.273
Il–10, pg/mL	24.276	25.123	25.631	23.053	0.627	0.652
AST, U/L	96.362	83.068	105.550	84.327	9.836	0.344
ALT, U/L	28.763	26.670	28.360	31.383	2.443	0.600
AST/ALT	3.361	3.135	3.759	2.736	0.300	0.141
ALP, U/L	54.990	46.710	52.343	48.392	3.805	0.425
LDH, U/L	1,148.382	1,100.962	1,144.188	990.893	59.743	0.454
CK, U/L	157.000	100.500	114.800	88.333	23.312	0.281

*IgA* Immunoglobulin A, *IgG* Immunoglobulin G, and *IgM* Immunoglobulin M, *IL-6* Interleukin 6, *IL-10* Interleukin 10, *AST* Aspartate aminotransferase, *ALT* Alanine aminotransferase, *ALB* Albumin, *LDH* Lactate dehydrogenase, *CK* Creatine kinase

^1^*HT* High frequency tongue rolling cows, *MT* Medium frequency tongue rolling cows, *LT* Low frequency tongue rolling cows, *CON* Control cows, no abnormal oral behaviour

^2^*SEM* Standard error of the mean

^a^^,^^b^Different superscript within a row means significant different (*P* < 0.05)

Spearman's correlation revealed the results of the correlation between tongue rolling frequency and serum stress indicators (Table 4). The results showed a moderate correlation trend between tongue rolling frequency and serum cortisol levels (*r* = 0.363, *P* = 0.081).

**Table 4:** Spearman correlation between tongue-rolling frequency and serum indicators

Items		TRF	COR	DA	GABA	5-HT	E	NE
TRF	*r*-value	1.000	0.363	0.243	0.252	0.226	0.095	0.227
	*P*-value		0.081	0.276	0.246	0.287	0.674	0.286
COR	*r*-value	0.363	1.000	0.299	0.424	0.236	0.551	0.312
	*P*-value	0.081		0.176	0.044	0.268	0.008	0.138
DA	*r*-value	0.243	0.299	1.000	0.487	0.607	0.561	0.531
	*P*-value	0.276	0.176		0.025	0.003	0.008	0.011
GABA	*r*-value	0.252	0.424	0.487	1.000	0.532	0.489	0.560
	*P*-value	0.246	0.044	0.025		0.009	0.024	0.005
5-HT	*r*-value	0.226	0.236	0.607	0.532	1.000	0.423	0.718
	*P*-value	0.287	0.268	0.003	0.009		0.050	0.000
E	*r*-value	0.095	0.551	0.561	0.489	0.423	1.000	0.497
	*P*-value	0.674	0.008	0.008	0.024	0.050		0.019
NE	*r*-value	0.227	0.312	0.531	0.560	0.718	0.497	1.000
	*P*-value	0.286	0.138	0.011	0.005	0.000	0.019	

*TRF* Tongue rolling frequency, *COR* Cortisol, *DA* Dopamine, *GABA* γ-hydroxybutyric acid, *5-HT* 5-hydroxytryptamine, *E* Epinephrine, *NE* Norepinephrine

### Ruminal fermentation indicators

We examined the pH and the contents of NH_3_-N and VFAs in rumen fluid (Table 5). Rumen fluid pH was significantly lower in MT group than in CON and HT group (*P* = 0.013). Among the rumen VFAs, the contents of total volatile fatty acids (*P* = 0.048), butyric acid (*P* = 0.007), valeric acid (*P* = 0.013), and isovaleric acid (*P* = 0.029) showed significant differences between the groups. With the increase of tongue rolling frequency, the above indicators showed a trend of first increasing and then decreasing. The LT group had the highest value among all groups. The contents of NH3-N were not significantly different among the four groups.

**Table 5:** Rumen fermentation parameters after feeding

Items	Experimental treatments^1^				SEM^2^	*P*-value
	HT	MT	LT	CON		
pH	6.132^a^	5.857^b^	6.055^ab^	6.249^a^	0.077	0.013
Ammonia-N, mg/dL	23.384	22.042	22.571	21.118	1.968	0.711
Acetate, mmol/L	64.964	69.187	75.917	67.648	3.811	0.246
Propionate, mmol/L	30.086	32.198	34.743	31.936	2.185	0.525
Acetic acid/propionic acid ratio	2.184	2.166	2.216	2.130	0.010	0.432
Butyrate, mmol/L	15.317^b^	21.437^a^	21.840^a^	16.993^b^	1.386	0.007
Isobutyrate, mmol/L	0.967	1.247	1.328	1.065	0.114	0.134
Valerate, mmol/L	2.035^b^	2.965^ab^	3.855^a^	2.238^b^	0.384	0.013
Isovalerate, mmol/L	1.573^b^	2.073^ab^	3.855^a^	1.714^b^	0.199	0.029
Total volatile fatty acids, mmol/L	114.941^b^	129.107^ab^	140.108^a^	121.594^b^	6.082	0.048

^1^*HT* High frequency tongue rolling cows, *MT* Medium frequency tongue rolling cows, *LT* Low frequency tongue rolling cows, *CON* Control cows, no abnormal oral behaviour

^2^*SEM* Standard error of the mean

^a^^,^^b^Different superscript within a row means significant different (*P* < 0.05)

### Composition and diversity of ruminal microbial community

**Fig. 3:**
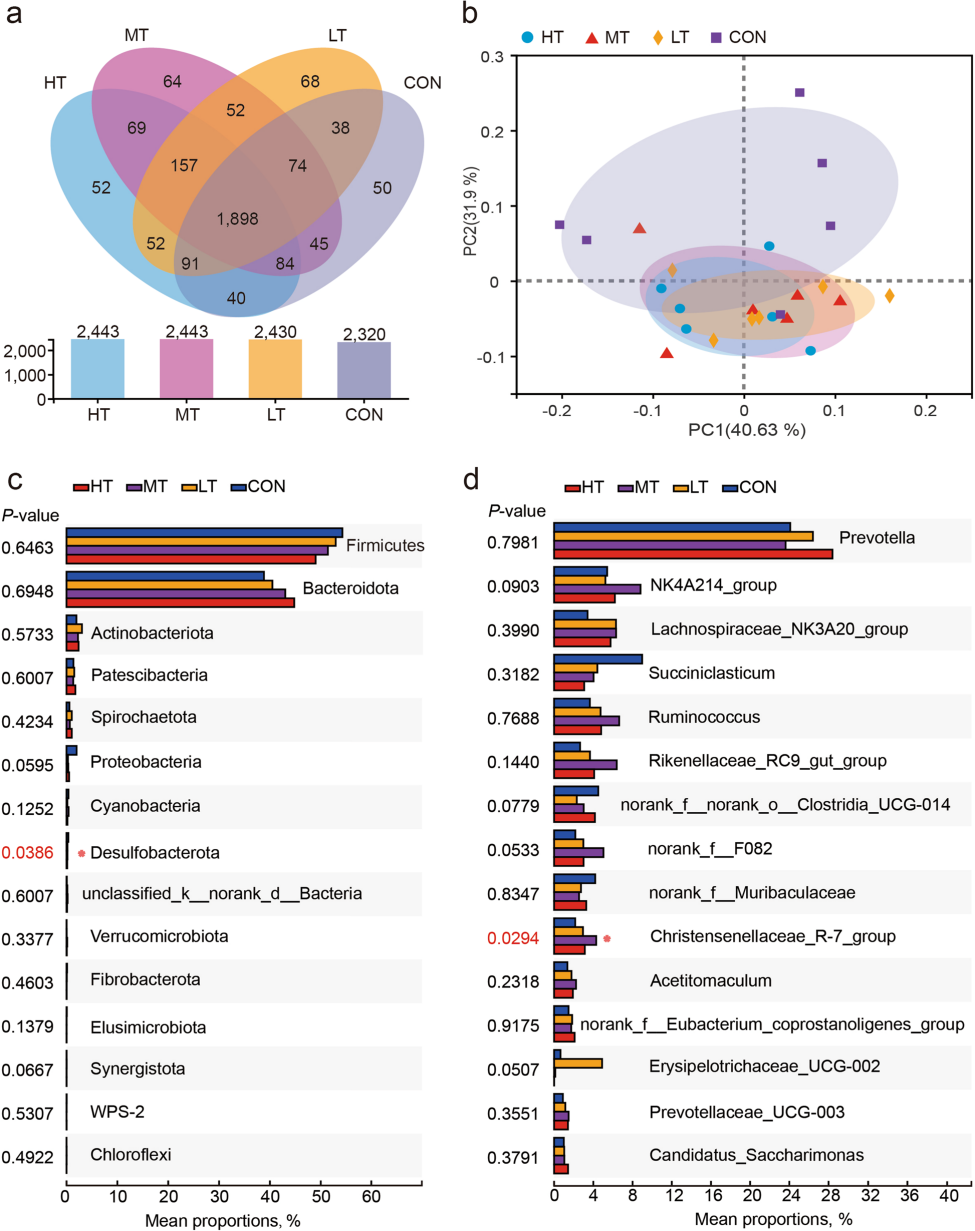
**a** A Venn plot of the shared OTUs among four cow groups with different frequency of tongue-rolling behaviour; **b** PCoA of phylum-level rumen microbiomes from four cow groups with different tongue-rolling frequency; **c** Top 15 abundant species at the phylum level; **d** Top 15 abundant species at the genus level. HT, cows with high tongue rolling frequency behaviour; MT, cows with medium tongue rolling frequency behaviour; LT, cows with low tongue rolling frequency behaviour; CON, cows without tongue rolling behaviour. * means *P* < 0.05

The total number of sequenced reads of the 24 rumen fluid samples ranged from 30,000 to 55,000, with an average length of over 420 nt. Finally, we obtained 2,840 OTUs,19 bacterial phyla, and more than 323 genera at 97% similarity. Richness, diversity, evenness, and coverage of the community were examined using the Chao1 and the ACE, Shannon, Simpson, and Good's coverage index, respectively. Table 6 showed all these diversities of indices. The Venn diagram depicts the unique or shared OTUs in groups CON, LT, MT, and HT (Fig. 3A). Only the OTUs of the tongue rolling groups were higher than that of the CON group, but not significant. Figure 3B shows that PCoA axis 1 and axis 2 at the phylum level accounted for 40.63% and 31.9% of the total variation, respectively. According to the result, the bacterial communities in group CON showed greater separation from HT, MT, and LT groups, while the HT, MT and LT groups could not be separated in the PCoA map. To ascertain the composition and relative abundance of the rumen microbiota, the OTUs were taxonomically annotated. Of the top 15 abundant bacterial phyla, Firmicutes and Bacteroidota had the highest abundance (more than 90%) (Fig. 3C). At the genus level, more than 323 genera were detected, in which *Prevotella* (mainly)*, NK4A214_group, Lachnospiraceae_NK3A20_group, Succiniclasticum, Ruminococcus, Rikenellaceae_RC9_gut_group, norank_f__norank_o__Clostridia_UCG-014, norank_f__F082, norank_f__Muribaculaceae, Christensenellaceae_R-7_group* predominated in ruminal fluid samples of HT, MT, LT, and CON group cows. Of the top 15 abundant genera, there were significant differences in the *Christensenellaceae_R-7_group* in the MT group (*P* = 0.029) and a trend toward significant differences between groups in the *NK4A214_group* (*P* = 0.090), *norank_f__norank_o__Clostridia_UCG-014* (*P* = 0.078), *norank_f__F082* (*P* = 0.053), and *Erysipelotrichaceae_UCG-002* (*P* = 0.051) (Fig. 3D).

**Table 6:** α diversity indicators of dairy cows' rumen bacteria

Items	Experimental treatments^1^				SEM^2^	*P*-value
	HT	MT	LT	CON		
OTUs	1,549.167	1,452.667	1,492.600	1,363.333	87.511	0.226
Chao1	1,845.363	1,746.976	1,792.381	1,663.072	96.754	0.316
ACE	0.009	0.012	0.010	0.012	0.002	0.477
Shannon	5.765	5.587	5.680	5.591	0.135	0.523
Simpson	0.009	0.011	0.010	0.012	0.002	0.114
Good’s coverage	1,863.024	1,773.607	1,818.990	1,669.978	92.717	0.228

^1^*HT* High frequency tongue rolling cows, *MT* Medium frequency tongue rolling cows, *LT* Low frequency tongue rolling cows, *CON* Control cows, no abnormal oral behaviour

^2^*SEM* Standard error of the mean

### Base behaviour recorded by the collar

**Fig. 4:**
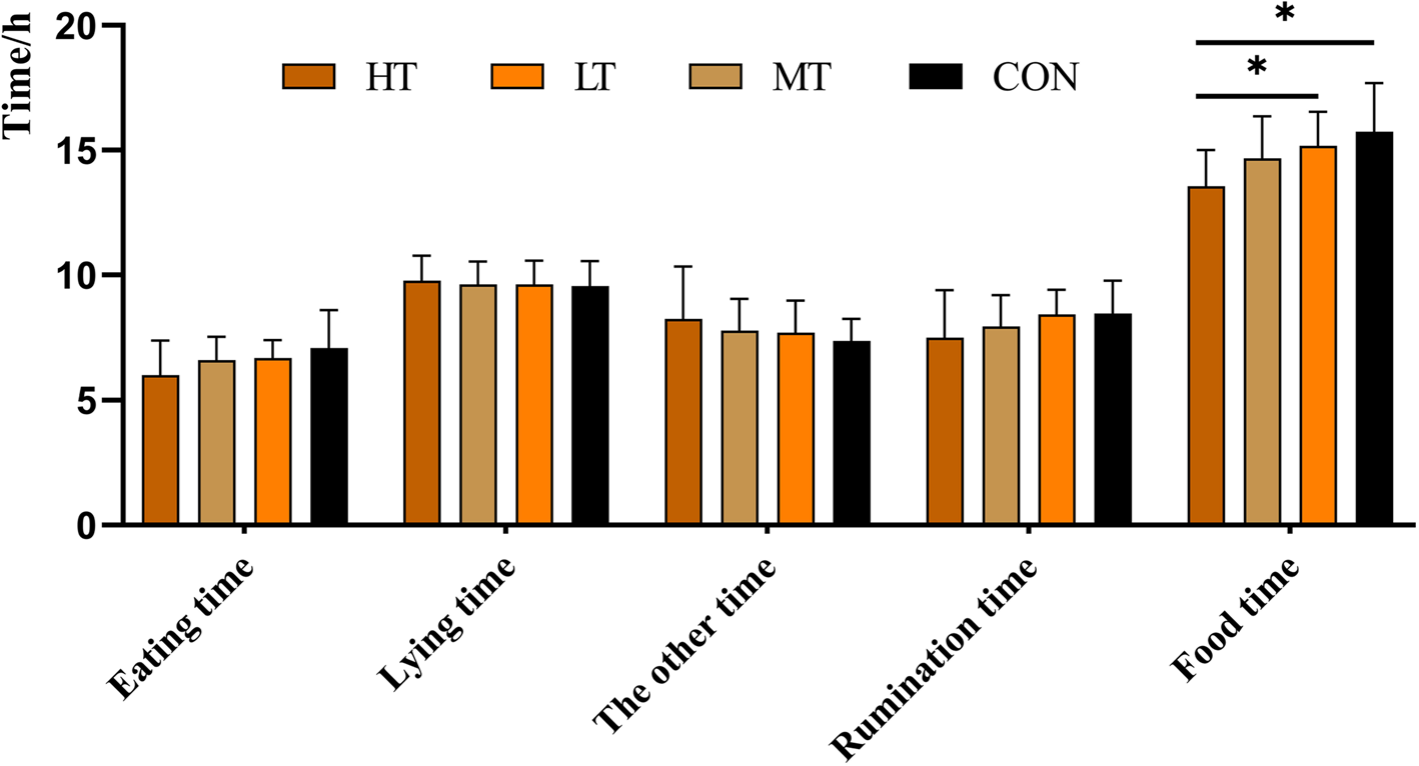
Model statistics of the general behaviours of dairy cows during the experiment procedure. The other time = 24 h - eating time - lying time, including idle standing time and milking time for cows, etc.; Food time = eating time + rumination time. HT, cows with high tongue rolling frequency behaviour; MT, cows with medium tongue rolling frequency behaviour; LT, cows with low tongue rolling frequency behaviour; CON, cows without tongue rolling behaviour. * means *P* < 0.05

Using the collar sensor, we recorded the time the cow spent on eating and lying over 24 h. We defined the time of the day other than eating and lying down as the other time, including idle standing time and milking time for cows, etc. Rumination time and food time (food time = eating time + rumination time) were recorded by the collar simultaneously. The results of the cows' behavioural times were shown in Fig. 4. The food time was significantly higher in the CON group than in the HT group (*P* = 0.035). And the LT group also had a significantly higher food time than the HT group (*P* = 0.049).

The correlations between tongue rolling frequency and time recorded were listed in Table 7. Spearman's correlation analysis showed that tongue rolling frequency had a moderate negative correlation with food time (*r* = −0.492, *P* = 0.015) and a moderate negative correlation trend with rumination time (*r* = −0.384, *P* = 0.064).

**Table 7:** Spearman correlation between tongue-rolling frequency and general activity time of cows

Items		TRF	Rumination time	Eating time	Food time	The other time	Lying time
TRF	*r*-value	1	−0.384	−0.277	−0.492	0.176	0.064
	*P*-value		0.064	0.190	0.015	0.410	0.767
Rumination time	*r*-value	−0.384	1	−0.079	0.743	0.152	−0.142
	*P*-value	0.064		0.715	0.000	0.479	0.508
Eating time	*r*-value	−0.277	−0.079	1	0.609	−0.772	−0.015
	*P*-value	0.190	0.715		0.002	0.000	0.944
Food time	*r*-value	−0.492	0.743	0.609	1	−0.397	−0.123
	*P*-value	0.015	0.000	0.002		0.055	0.565
The other time	*r*-value	0.176	0.152	−0.772	−0.397	1	−0.624
	*P*-value	0.410	0.479	0.000	0.055		0.001
Lying time	*r*-value	0.064	−0.142	−0.015	−0.123	−0.624	1
	*P*-value	0.767	0.508	0.944	0.565	0.001	

*TRF* Tongue rolling frequency, The other time = 24 h − eating time − lying time, including idle standing time and milking time for cows, etc.; Food time = eating time + rumination time

## Discussion

The cow herd grouping in this study was based on the scan sampling behaviour observation method, and although all moments of tongue-rolling behaviour in cows could not be completely recorded under this observation method, it did not affect the actual selection results. And, the study was able to identify the maximum range of tongue-rolling frequency in cows in the barn through 126 scan sampling behaviour observations and was able to determine the extent of tongue-rolling behaviour expressed in cows. We collected the background information, recorded the general behaviour time and tested the ruminal fluid samples and serum samples of cows with different tongue-rolling frequencies. By correlating the results with tongue-rolling frequency, the pattern of their correspondence was found, which would provide more insight into the relationship between cows' tongue-rolling behaviour with their general behaviour and physiological health. These changes were most likely due to long-term chronic environmental stress, as the level of stress corresponded to the expression of stereotypic behaviour.

### The relationship between tongue rolling frequency and body stress and immunity

When animals are stressed, the HPA axis and LC-NE system are further activated to promote the production of glucocorticoids and catecholamine hormones by the adrenal cortex [13]. This response helps to mobilize body energy and maintain the stability of the internal environment. Different stressful stimuli (social or physical) activate the HPA axis and LC-NE system through different mechanisms [13, 35]. Monoamine neurotransmitters, especially dopamine, 5-hydroxytryptamine, and norepinephrine, played an important role in the activation and modulation of the HPA axis and LC-NE system. Previous research found that corticosteroids may act differently during the early formative and fully developed stages of stereotypic behaviour [36, 37]. In this study, the levels of the above indicators in dairy cows had similar changes with the increasing of tongue rolling frequency, indicating that there might be an interaction between tongue-rolling behaviour and HPA axis and LC-NE system response, but the causal relationship was still uncertain.

Repeated or long-term exposure to pressure would lead to changes in HPA axis and LC-NE axis function and pressure responses [13, 37]. High stress level and increased corticosteroid secretion enhance the acquisition and expression of stereotyped behaviours, while already-formed stereotypes might reduce corticosteroid levels [38]. Therefore, it was meaningful to perform a cross-sectionally study to investigate whether the high-frequency tongue-rolling behaviour reduces cortisol levels and whether brief spikes in stress levels precede stereotyped behaviours.

Interestingly, we observed that serum cortisol levels had a moderate positive correlation with tongue rolling frequency in dairy cows, which did not decline adaptively with elevated stereotype frequency levels. In a study on stereotypically behaving horses with chronic stress, researchers observed increased or maintained high HPA axis responses in these animals when facing new stress stimuli [5]. The findings were consistent with the results in stereotyped horses. The high cortisol levels in high-frequency tongue-rolling cows might result from the accumulation of chronic stress [39]. This result was inconsistent with our original hypothesis, indicating that tongue rolling is not an adaptive behaviour, and its high-frequency expression may not divert self-stress, nor stabilize or reduce the level of stress hormones in the body. It was worth noting that passively responding animals had higher HPA axis reactivity and cortisol levels than actively responding animals under the same environmental stress stimulus [40]. The higher levels of cortisol suggested that these cows with high-frequency behaviour have a higher level of stress response, suggesting that the frequency of tongue-rolling behaviour could be seen as a behavioural indicator of stress level.

Furthermore, tongue-rolling behaviour might be associated with neurological dysfunction and related mood disorders. Anxiety disorders, post-traumatic stress disorder, and depression were characterized by excessive stress, anxiety, and overreacted HPA axis, which increases the levels of cortical stress hormones [41]. This increase in hormone levels created a state of readiness in which the body performs some “alert responses” [42]. HPA-axis hyperactivity and inflammation were thought to be prominent phenotypes in the aetiology of depression or major depressive disorder. Higher levels of cortisol were significantly associated with persistent depressive symptoms. When cows were repeatedly exposed to a pressure environment, the HPA axis changed similar to depression symptoms [43]. In this study, as the serum cortisol levels increased in cows with stereotyped behaviour, changes in serum inflammatory markers IgA and IgG from MT to HT groups were consistent with the representation of depression. Accordingly, it could be speculated that the high frequency of tongue-rolling behaviour may be a potential manifestation of depressed cattle. In the range recorded in this study, we considered that the higher the frequency of tongue rolling, the deeper the degree of depression.

### The relationship between tongue rolling frequency and rumen fermentation

Saliva and rumen feed were mixed during rumination, which kept the pH level and fermentation environment stable. Cows fed high-fiber feed had higher rumination and total chew [44]. Adding high-fiber feed (gluten feed) to the diet increased the frequency of feed intake, reduce the amount of feed intake, and prolong the feeding time. And reduced intake of roughage feed could reduce rumination time and further reduce the pH [45]. Low rumen pH (below 6) would inhibit the growth of rumen pH sensitive cellulolytic bacteria, affect the digestion of cellulose [46], and increase the risk of subacute ruminal acidosis. According to studies, cow rumination occurred more frequently when the animal was lying down. Compared to standing rumination, cows were more inclined to ruminate while lying down [47]. Herrbaut found that the lying time was negatively correlated with time pH < 6 in the rumen [48]. In this study, the pH of cows in the MT group was less than 6, but the lying time of cows in the MT group was not significantly increased. And the pH of group HT did not change with the decrease of rumination time like that of the MT group. On the one hand, this result might be because the pH detection in this study was instant, and the duration of pH < 6 is not measured. Since the ruminal pH potentially varies throughout the day, one-time pH measurements cannot adequately reflect ruminal pH dynamics and persistence [49]. On the other hand, changes in gastrointestinal functional status might affect hormone levels through the HPA axis [50], while gastrointestinal discomfort could manifest as oral stereotypical behaviour [7]. Tongue-rolling behaviour might act as an alternative activity to compensate for decreased rumination activity, by producing more saliva to buffer the pH decrease and alleviate the rumen discomfort resulted from high concentrate feeding [44]. This study found that HT cows had higher rumen pH compared to those in the MT cows, suggesting increased saliva secretion due to tongue rolling. This observation indicated that tongue-rolling behaviour may stimulate more saliva secretion, and thus serve as a beneficial mechanism for maintaining rumen health in high-concentrate-fed cows [7]. The change in rumen pH is in line with the changes in hormone levels secreted by the HPA axis and LC-NE system. Therefore, we inferred that there might be a correlation between tongue-rolling behaviour, gastrointestinal comfort, and hormone levels regulated by the HPA axis and LC-NE system [7, 13].

The pH of the rumen played a critical role in the effectiveness of rumen fermentation caused by microorganisms [51]. It accurately reflected the internal environmental conditions and level of rumen fermentation [52]. As carbohydrate fermentation in the rumen increases, rumen pH decreases, which favors the growth of amylolytic microbial growth over cellulolytic microbial growth [44]. Previous studies found that cows with high-frequency tongue-rolling behaviour (similar to the HT group in this study) had lower ruminal pH and VFAs, and richer OTU abundance of ruminal bacterial flora, including increased Bacteroidota and decreased Firmicutes at the phylum level, and increased *Prevotella* at the genus level [5]. The difference in Bacteroides favoured the rumen to decompose concentrated feed. It was hypothesized that the tongue-rolling behaviour influenced the feed intake process of cows, favouring the feeding of concentrate feeds. In the present study, the differences in rumen pH and rumen bacteria OTU indicators in cattle from the LT, MT, and HT groups were in line with previous studies. The presence of rumen cocci (*Prevotella*, *NK4A214_group*, and *Lachnospiraceae_NK3A20_group*) at the genus level was significantly increased in the 3 tongue-rolling groups, implying an increased starch catabolic capacity.

In this study, the changes in ruminal VFA indicators were opposite between the LT and MT groups. Rumen VFAs were produced during the final breakdown of carbohydrates by rumen bacteria. VFA levels in rumen fluid and the relative proportion of each VFA species depend on the composition and proportions of the substrate, the presence of specific rumen microbial species, the rumen's pH, and the feeding pattern [53–55]. Under excessive tongue rolling, the excessive secretion of saliva could actually increase rumen satiety [56], which in turn reduced feed intake and rumination. From the behaviour duration recorded on the collar, it could be seen that there was a negative correlation between tongue rolling behaviour and food time. Excessive tongue rolling reduced the feed intake. At this point, the saliva buffering effect produced by high-frequency tongue rolling in cows could only regulate the discomfort of acidic fermentation without substantial changes in the structure of fermentation content and feed intake. This resulted in lower VFA levels in the HT and MT groups of cows. Therefore, excessive tongue rolling did not compensate for rumen fermentation, resulting in a decrease in related fermentation indicators. Additionally, the individual differences within groups could not be ignored. In order to find a more precise model, further study considering a larger sample size is ongoing.

### Relationship between tongue rolling frequency and general activity

Disease state of cows can affect their activity levels, behavioural patterns, or both [57–60]. The duration and intensity of unitary behaviours and movements of cattle help predict their calving, estrus, lameness, and disease [19]. Numerous researchers have investigated factors influencing cattle behaviour categorization for more accurate monitoring of cattle health [61, 62].

Lying time and rumination time were two critical indicators of the productive performance and welfare of dairy cows. Their physical health could be negatively affected if lying time is compromised. For example, shorter lying times may be a risk factor for lameness in grazing cows [63], affecting the function of the pituitary-adrenal axis, and leading to increased chronic stress [64]. According to previous research, cows with high-frequency tongue-rolling behaviour had longer lying and drinking time, and were better at coping with stressful situations [5], which was consistent with the HT group in this study. However, there was no strong linear correlation between the frequency of tongue-rolling behaviour and lying time in this study. Compared with the differences in lying time and stress hormone levels among the four groups, we supposed that there was a multitude of factors that contributed to the lying time, hence further causal relationships between indicators need to be uncovered and explored.

The food time recorded by the collar included the processes of feeding and rumination, both of which were related to the tongue activity of cows. In this study, we noticed that the tongue-rolling frequency of cows had a moderately negative relationship with their rumination time and food time. We speculated that the occurrence of tongue-rolling behaviour might be associated with food behaviour, and the frequency of tongue rolling increased as food time decreases. When fed a higher proportion of concentrate, ruminants would spend less time ruminating [65]. Branger et al. hypothesised that the oral stereotypes might be a vacuum or redirected behaviour resulting from insufficient tongue activity due to insufficient feed intake and ruminate time [7]. Tongue-rolling behaviour in this study was also consistent with this inference. Oral manipulation of feed with the tongue is a natural behavioural need of cows [66], and oral stereotyped behaviours such as tongue rolling are redirected behaviours when oral manipulation of feed is lacking. In addition, whether the tongue-rolling behaviour interferes with the cow's natural foraging activities needs further investigation.

## Conclusion

The frequency of tongue-rolling behaviour in dairy cows might be correlated to their stress level. Tongue-rolling behaviour was more likely to occur when the cows lack natural oral foraging activities. The degree of tongue-rolling behaviour might be related to feeding components and rumen fermentation.

## Data Availability

The data analyzed during the current study are available from the corresponding author on reasonable request.

## Abbreviations

5-HT: 5-Hydroxytryptamine
ALB: Albumin
ALT: Alanine aminotransferase
AST: Aspartate aminotransferase
CK: Creatine kinase
CON: Control group
COR: Cortisol
DA: Dopamine
E: Epinephrine
GABA: γ-Hydroxybutyric acid
HPA: Hypothalamic–pituitary–adrenal
HSP-70: Heat shock protein 70
HT: High tongue rolling frequency group
IgA: Immunoglobulin A
IgG: Immunoglobulin G
IgM: Immunoglobulin M
IL-6: Interleukin 6
IL-10: Interleukin 10
IMU: Inertial measurement units
LDH: Lactate dehydrogenase
LT: Low tongue rolling frequency group
MT: Medium tongue rolling frequency group
NE: Norepinephrine
NH_3_-N: Ammonia-N
OTUs: Operational taxonomic units
PABAK: Prevalence-adjusted, bias-adjusted kappa
RDP: Ribosomal database project
TMR: Total mixed rations
TP: Total protein
VFA: Volatile fatty acids
